# R2-ISS staging combined with circulating plasma cells improves risk stratification for newly diagnosed multiple myeloma: a single-center real-world study

**DOI:** 10.1007/s00277-024-05806-9

**Published:** 2024-07-03

**Authors:** Bin Chu, Yu-tong Wang, Shan Gao, Lei Shi, Min-qiu Lu, Li-juan Fang, Qiu-qing Xiang, Yuan Chen, Meng-zhen Wang, Li-fang Wang, Kai Sun, Jing Yang, Fangfang Duan, Li Bao

**Affiliations:** 1 Department of Hematology, Beijing Jishuitan Hospital, Capital Medical University, No. 31 East Xinjiekou Street, Xicheng District, Beijing, 100035 China; 2 Houston Methodist Cancer Center, Houston Methodist Research Institute, Houston Methodist Hospital, Houston, TX 77030 USA; 3 Clinical Epidemiology Research Center, Beijing Jishuitan Hospital, Capital Medical University, Beijing, 100035 China

**Keywords:** Multiple myeloma, Circulating plasma cell, Overall survival, Prognosis, R2-ISS stage

## Abstract

We aimed to evaluate if circulating plasma cells (CPC) detected by flow cytometry could add prognostic value of R2-ISS staging. We collected the electronic medical records of 336 newly diagnosed MM patients (NDMM) in our hospital from January 2017 to June 2023. The median overall survival (OS) for patients and R2-ISS stage I-IV were not reached (NR), NR, 58 months and 53 months, respectively. There was no significant difference in OS between patients with stage I and patients with stage II (*P* = 0.309) or between patients with stage III and patients with stage IV (*P* = 0.391). All the cases were re-classified according to R2-ISS stage and CPC numbers ≥ 0.05% (CPC high) or<0.05% (CPC low) into four new risk groups: Group 1: R2-ISS stage I + R2-ISS stage II and CPC low, Group 2: R2-ISS stage II and CPC high + R2-ISS stage III and CPC low, Group 3: R2-ISS stage III and CPC high + R2-ISS stage IV and CPC low, Group 4: R2-ISS stage IV and CPC high. The median OS were NR, NR, 57 months and 32 months. OS of Group 1 was significantly longer than that of Group 2 (*P* = 0.033). OS in Group 2 was significantly longer than that of Group 3 (*P* = 0.007). OS in Group 3 was significantly longer than that of Group 4 (*P* = 0.041). R2-ISS staging combined with CPC can improve risk stratification for NDMM patients.

**Supplementary Information:**

The online version contains supplementary material available at 10.1007/s00277-024-05806-9.

## Introduction

Multiple myeloma (MM) is a malignancy characterized by highly heterogeneous clinical features, biology, and outcomes [1, 2]. Currently, R-ISS staging is the primary method used for prognostic evaluation and stratified treatment [3]. R-ISS staging evolved from ISS staging [4], which initially required only two parameters—albumin and β2-microglobulin. The inclusion of lactate dehydrogenase (LDH) and high-risk cytogenetic abnormalities in the R-ISS system has led to improved prognostic stratification compared to ISS staging. However, R-ISS staging resulted in a substantial population classified as stage II and contributed to increased survival heterogeneity in stage II. To address the challenge of insufficient differentiation in outcomes within the intermediate-risk population (R-ISS stage II), the recent retrospective analysis conducted by the European Myeloma Network (EMN) resulted in the development of a new risk model known as the R2-ISS System [5]. This system demonstrated favorable risk distribution among patients participating in clinical trials. However, there were limited researches verifying the applicability of the R2-ISS staging system in real-world scenarios.

In several retrospective studies [6–10], no significant difference in survival was observed between patients with R2-ISS stage I and stage II, while different conclusions were drawn on whether there were differences in survival between patients with stage III and stage IV. Therefore, the effectiveness of R2-ISS staging in predicting clinical outcomes in the real world needs to be further verified.

Circulating plasma cells (CPC) are plasma cells found in the peripheral blood circulation of MM patients. The optimal clinical relevance of CPC is evident in primary plasma cell leukemia (PCL) whose median survival is approximately less than two years. The amount of plasma cells for defining PCL has decreased from ≥ 20% [11] to ≥ 5% by morphology in blood smears [12]. Recent clinical studies have identified PCL-like patients with as little as 2% CPC detected by flow cytometry [13]. All of these studies suggest that even small amounts of CPC have prognostic value. Several prior studies have verified that CPC numbers can serve as a risk factor for poor prognosis in NDMM patients [14–18].

This study retrospectively analyzed the clinical data of NDMM patients treated with new drugs in northern China to investigate whether CPC numbers could be employed as a biological marker to further enhance the efficacy of the R2-ISS staging system in prognostic stratification in the real world study.

## Materials and methods

### Case data

We collected the electronic medical records of 357 consecutive patients diagnosed NDMM in our hospital from January 2017 to June 2023. The diagnosis of MM [19] and treatment response assessment followed established guidelines [20]. The study protocol received approval from the Institutional Ethics Committee of our hospital (ethical batch number K2023-014-00), and all patients provided written informed consent.

Observational indicators. Patient clinical data, encompassing gender, age, blood cell count, biochemistry, disease stage and risk stratification, bone marrow and blood plasma cell analysis, immunophenotyping, cytogenetics and treatment regimens were systematically collected. Presence of any one or more chromosomal abnormalities, such as 1q21+, del(17p), t(4;14), t(14;16), or t(14;20), were defined as high-risk cytogenetic abnormalities [21].

### Flow cytometry analysis

Immunophenotyping was performed using an DxFLEX eight-colour flow cytometer (Beckman company). Whole erythrocyte-lysed BM samples were stained using the following combinations: tube 1: CD138-APC / CD38-AF700 / CD45-PerCP / CD269-PE / CD27-FITC; tube 2: CD138-APC / CD38-AF700 / CD45-PerCP / cKappa-FITC / cLamda-PE / CD56-APC-CY7 / CD19-ECD. To assess the surface antigens, an aliquot of approximately 1 × 10^6^ cells was labelled and antibodies in accordance and the manufacturer’s recommendations (BD Biosciences). The cells were then washed and phosphate-buffered saline and suspended in 1% paraformaldehyde. Signal acquisition was performed and at least 1 × 10^6^ events per sample. Malignant plasma cells were categorized according to the recommendation of EMN [22]. The expression of an antigen was considered positive when ≥ 20% of the plasma cells expressed it. The CPC was reported by the clonal plasma cell ratio and it was considered no CPC detected when the ration was < 0.01% (100cells/10^6^events).

### Fluorescence in situ hybridization (FISH) analysis

The probes detected by FISH corresponded to sites 1q21, 17p13 (P53), 14q32/11q13 (CCND1/IGH), 4p16/14q32 (FGFR3/IGH) and 14q32/16q23 (IGH/MAF). Bone marrow plasma cells sorted by CD138 monoclonal antibody were processed by permeabilization, fixation, incubation on slides at 37 °C in fresh 2 × SSC (pH 7.0) solution for 1 h, gradient ethanol dehydration, and drying at room temperature. Then, after DNA probe denaturation, hybridization, washing and re-dyeing, the cells were viewed under a microscope. Bone morrow cells from 20 patients and non-malignant hematological disease were selected as the control group for the establishment of the threshold, and the average number of positive cells of each probe in the control group ± 2 times of standard deviation was used as the threshold. The cutoff value was 8% for 17p13 and 6% for the remaining targets.

### Follow-up

Follow-up was conducted by reviewing electronic medical records and telephone calls, persisted until October 1, 2023 or death with a median follow-up of 32 (IQR 12.70–52.00) months. Endpoints included overall survival (OS) and disease progression-free survival (PFS). OS was defined from diagnosis to death from any cause or the last follow-up date. PFS was defined from treatment initiation to the first progression of disease or death from any cause, whichever occurred first. During the follow-up period, 8 patients were lost so 328 patients were finally included in the survival analysis.

### Statistical analysis

Statistical analysis and visualization were performed using SPSS 21.0 and GraphPad Prism 9.0 and R4.2.3 statistical software. Non-normally distributed continuous variables were expressed as median (range), and categorical variables as absolute numbers and relative frequencies. Receiver operating characteristics (ROC) curve analysis was employed to determine the optimal CPC cutoff value. Patients were categorized into two groups based on the CPC threshold, and differences in continuous numerical variables between groups were tested using the Mann-Whitney U test, while categorical variables were assessed for differences between groups. Survival analysis utilized the Kaplan-Meier and log-rank method, and COX regression was applied for multivariate survival analysis. Two-sided p-values < 0.05 were deemed statistically significant.

## Results

### The baseline characteristic and front-line treatment

In our hospital the electrical medical records showed there were totally 357 consecutive NDMM cases from January 2017 to June 2023, Among the 357 cases, there were 5 cases (1.4%) who were defined as primary plasma cell leukemia at the time of diagnosis, 6 cases (1.7%) who had no CPC data, 2 cases (0.5%) who did not receive novel agents, 8 cases (2.2%) who had no R2-ISS staging data. These 21 cases were not suitable for the purpose of this study and were excluded. Finally, 336 cases (94.1% of 357 cases) were included in the study. And the baseline characteristics were shown in Table 1.

**Table 1:** Base-line characteristics of patients with newly diagnosed multiple myeloma patients (*n* = 336)

Parameter	Median value (range) or number (%)
Age (year)	62 (27–88)
<65 year	194 (57.7%)
≥ 65 year	142 (42.3%)
Male	212 (63.1%)
Female	124 (36.9%)
*M protein*	
IgG	152 (45.2%)
IgA	80 (23.8%)
IgD	7 (2.1%)
light chain	86 (25.6%)
non-secretory	11 (3.3%)
BMPC (%)	29.5 (0–97)
Calcium (mmol/L)	2.37 (1.74–4.60)
Creatinine (µmol/L)	75 (33–890)
Haemoglobin (g/L)	103 (42–167)
LDH (IU/L)	177 (28-2224)
β2-MG (mg/L)	4.85 (1.16–47.02)
Albumin (g/L)	38.0 (19.0-51.9)
*ISS stage*	
I	112 (33.3%)
II	91 (27.1%)
III	133 (39.6%)
*R-ISS stage*	
I	82 (24.4%)
II	167 (49.7%)
III	87 (25.9%)
*R2-ISS stage*	
I	56 (16.7%)
II	65 (19.3%)
III	168 (50.0%)
IV	47 (14.0%)
*FISH result*	
high risk	181 (53.9%)
standard risk	155 (46.1%)
*Induction Regimens*	
PI	186 (55.4%)
IMiD	5 (1.5%)
PI + IMiD	123 (36.6%)
Daratumumab	22 (6.5%)
*Front-line ASCT*	
Yes	124 (36.9%)
No	212 (63.1%)
*Front-line maintenance*	
Lenalidomide	124 (36.9%)
Ixazomib	100 (29.8%)
Lenalidomide + Ixazomib	32 (18.5%)
*Second-line maintenance*	80 (23.8%)
Daratumumab + Dexamethasone	33 (9.8%)
Pomalidomide + Dexamethasone	14 (8.0%)
Ixazomib + Dexamethasone	10 (2.1%)
Lenalidomide + Ixazomib + Dexamethasone	23 (3.9%)

*Note* BMPC, bone marrow plasma cells; FISH, fluorescence in situ hybridization; PI, proteasome inhibitors; IMiD, immunomodulatory drugs; LDH, lactase dehydrogenase; β2-MG, beta2 microglobulin; ASCT, auto stem cell transplantation

### CPC level and its effect on treatment response and survival

CPC was detectable in 188 (56%) patients with a median proportion of 0.10% (IQR 0.01 – 4.94%). In another 148 (44%) patients CPC was not detected. The ROC analysis revealed an optimal cut-off associated with worse OS to be 0.05% (500 cells/10^6^ events), showing a sensitivity of 58.06% and specificity of 100%. Utilizing this cut-off, patients were categorized into two groups: CPC < 0.05% group (CPC low, *n* = 209) with CPC number 0.00% (IQR 0.00 – 0.01%); CPC ≥ 0.05% group (CPC high, *n* = 127) with CPC number 0.21% (IQR 0.10 – 0.93%).

In ISS stage I-III, the proportion of patients with CPC high in each stage was 16%, 38% and 56%, and the proportion of patients with CPC low in each stage was 84%, 62% and 44%. In R-ISS stage I-III, the proportions of patients with CPC high in each stage were 17%, 36% and 60%, and the proportions of patients with CPC low in each stage were 83%, 64% and 40%. In R2-ISS stage I-IV, the proportions of patients with CPC high in each stage were 12%, 20%, 44% and 70%, and the proportions of patients with CPC low in each stage were 88%, 80%, 56% and 30% (supplementary Fig. 1).

Compared with CPC low group, the CPC high group were older (61 vs. 65 years, *P* = 0.029), exhibited a higher percentage of bone marrow plasma cells (18% vs. 43.5%, *P* = 0.003), a higher incidence of anemia (36.8% vs. 58.3%, *P* = 0.037) and renal insufficiency (9.6% vs. 18.1%, *P* = 0.031), as well as higher percentage of patients with LDH ≥ 250IU/L (14.8% vs. 20.5%, *P* = 0.021). Additionally, in the CPC high group a higher proportion of patients demonstrated high risk FISH results (44% vs. 71%, *P* = 0.001), ISS stage III (28.2% vs. 58.2%, *P* = 0.001), R-ISS stage III (16.7% vs. 41.0%, *P* = 0.001) and R2-ISS stage IV (6.7% vs. 26.0%, *P* = 0.001). The CPC high group had a lower proportion of patients achieving a deep induction treatment response (CR + VGPR) (40.2% vs. 60.8%, *P* = 0.029) and a higher proportion of patients experienced disease progression after induction therapy (17.3% vs. 1.4%, *P* = 0.013) (Table 2).

**Table 2:** Clinical characteristics comparison of patient groups with different levels of clonal circulating plasma cells

Parameter	Total (*n* = 336)	CPC low (< 0.05%) (*n* = 209)	CPC high (≥ 0.05%) (*n* = 127)	*P* value
Age (year) median (range)	62 (27–88)	61 (27–82)	65 (37–88)	0.029
Sex, n (%)				0.714
Male	212 (63.1)	129 (61.7)	83 (65.4)	
Female	124 (36.9)	80 (38.3)	44 (34.6)	
eGFR<40 ml/min, n (%)	43 (12.8)	20 (9.6)	23 (18.1)	0.031
HGB<100 (g/L)	151 (44.9)	77 (36.8)	74 (58.3)	0.037
Calcium>2.75 (mmol/L)	40 (11.9)	22 (10.5)	18 (14.2)	0.747
Bone marrow plasma cells (%)	29.5 (0–97)	18 (0–90)	43.5 (0–97)	0.003
LDH>250 IU/L, n (%)	57 (17.0)	31 (14.8)	26 (20.5)	0.021
FISH, n (%)				0.001
Standard risk	155 (46.1)	117 (56.0)	38 (29.9)	
High risk	181 (53.9)	92 (44.0)	89 (70.1)	
ISS stage, n (%)				0.001
I	112 (33.3)	94 (45.0)	18 (14.2)	
II	91 (27.1)	56 (26.8)	35 (27.6)	
III	133 (39.6)	59 (28.2)	74 (58.2)	
R- ISS stage, n (%)				0.001
I	82 (24.4)	68 (32.5)	14 (11.0)	
II	167 (49.7)	106 (50.7)	61 (48.0)	
III	87 (25.9)	35 (16.7)	52 (41.0)	
R2-ISS stage, n (%)				0.001
I	56 (16.7)	49 (23.4)	7 (5.5)	
II	65 (19.3)	52 (24.9)	13 (10.2)	
III	168 (50.0)	94 (45.0)	74 (58.3)	
IV	47 (14.0)	14 (6.7)	33 (26.0)	
Treatment, n (%)				0.747
PI	186 (55.4)	111 (53.1)	75 (59.1)	
IMiD	5 (1.5)	5 (2.4)	0 (0.0)	
PI + IMiD	123 (36.6)	79 (37.8)	44 (34.6)	
Daratumumab	22 (6.5)	14 (6.7)	8 (6.3)	
Induction response, n (%)				0.039
CR + VGPR	178 (53.0)	127 (60.8)	51 (40.2)	
PR	72 (21.4)	36 (17.2)	36 (28.3)	
MR	13 (3.9)	10 (4.8)	3 (2.4)	
SD	23 (6.8)	15 (7.2)	8 (6.3)	
PD	25 (7.4)	3 (1.4)	22 (17.3)	
No evaluation	25 (7.4)	18 (8.6)	7 (5.5)	

*Note* FISH, fluorescence in situ hybridization; PI, proteasome inhibitors; IMiD, immunomodulatory drugs; LDH, lactase dehydrogenase; CR, complete remission; VGPR, very good partial remission; PR, partial remission; MR, minor remission; SD, stable disease; PD, progression of disease

**Fig. 1:**
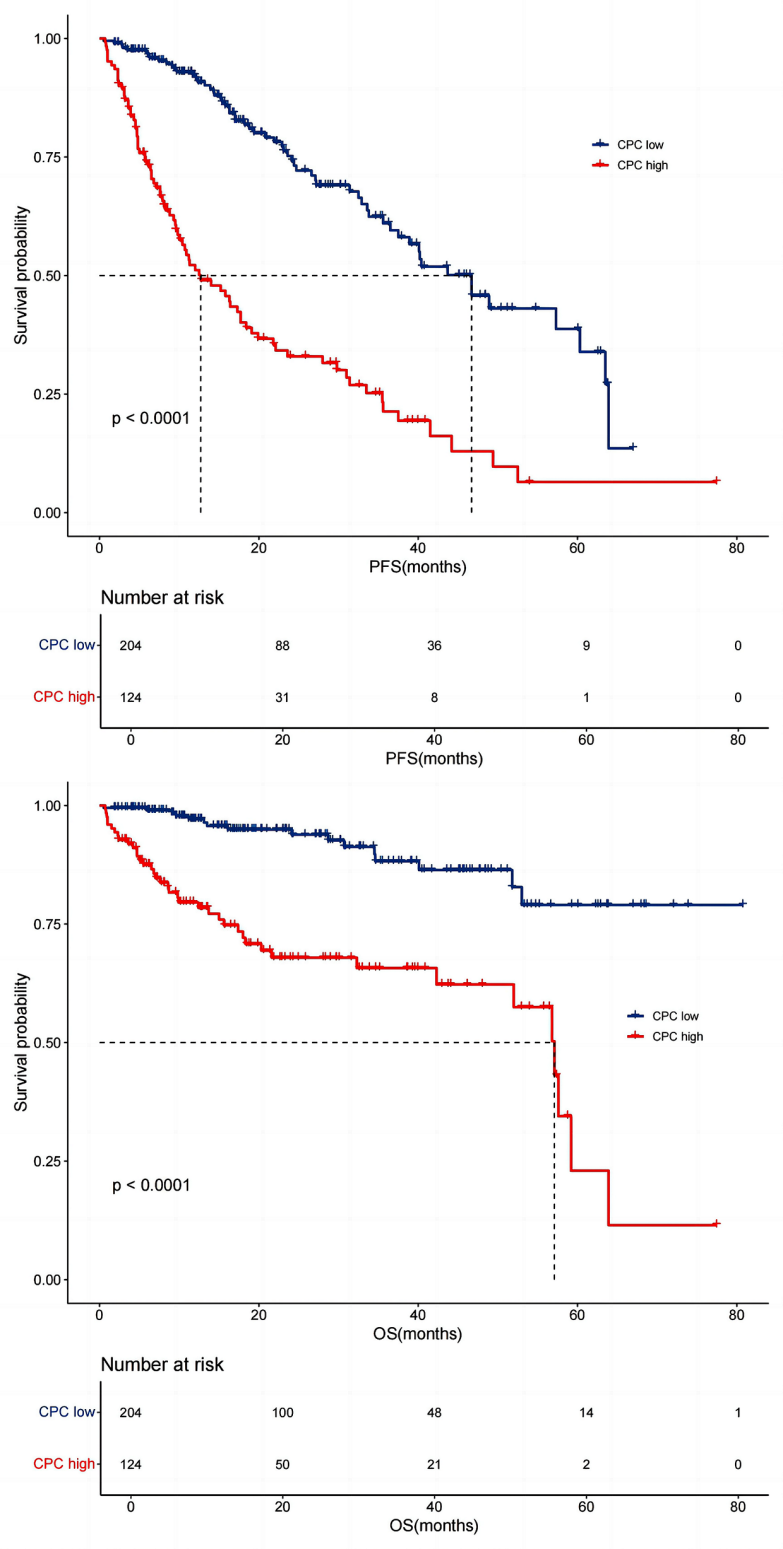
Circulating plasma cell (CPC) number and survival of newly diagnosed multiple myeloma (NDMM) patients. Median PFS in the CPC low (< 0.05%) and CPC high (≥ 0.05%) groups were 46 and 15 months, respectively. The median OS were not reached in the CPC low group and 58 months in the CPC high group

The survival analysis results revealed that the median PFS in the CPC low (*n* = 209) and CPC high (*n* = 127) groups were 46 months (IQR 11.66–57.00) and 15 months (IQR 10.46–35.60), respectively. The median OS was not reached in the CPC low group and 58 months (IQR 26.10–60.30) in the CPC high group. PFS in the CPC high group was significantly shorter than that in the CPC low group (*P*<0.0001). OS in the CPC high group was also significantly shorter than that in the CPC low group (*P*<0.0001) (Fig. 1).

**Fig. 2:**
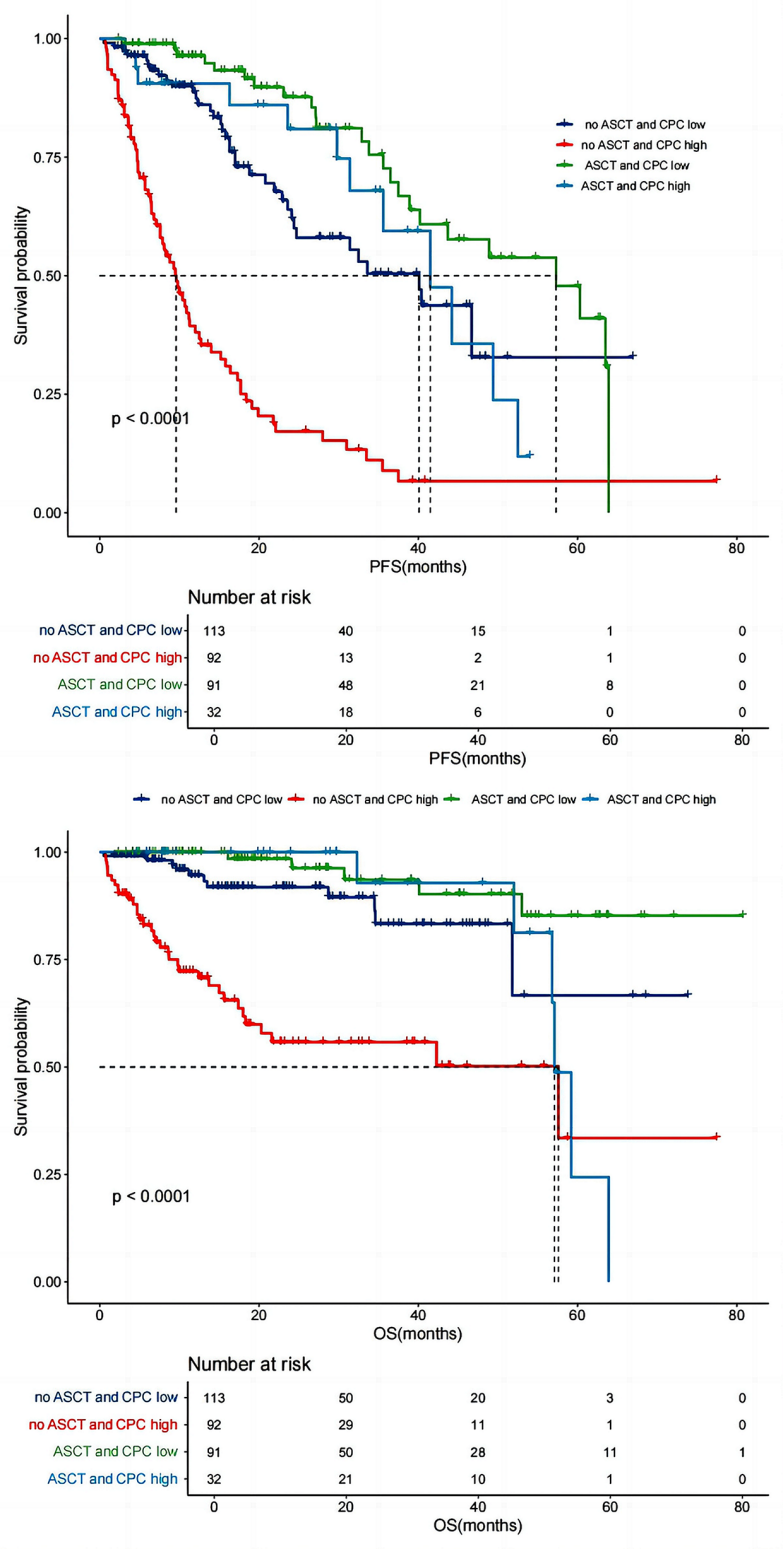
Effect of CPC level on the survival of NDMM patients in the transplant-eligible or ineligible subgroups. In the patients who received ASCT, the median PFS and OS of the CPC high subgroup were 42 months and 57 months, the median PFS and OS of the CPC low subgroup were 58 months and not reached. In the patients who did not receive ASCT, the median PFS and OS of the CPC high subgroup were 10 months and 58 months, the median PFS and OS of the CPC low subgroup were 40 months and not reached

To observe the impact of CPC count on prognosis in the transplant-eligible or ineligible subgroups, we regrouped the patients into 4 groups.The results showed that in the patients who received ASCT, the PFS and OS of the CPC high subgroup (*n* = 32) were significantly shorter than those of the CPC low subgroup (*n* = 91) (PFS:42 months vs. 58 months, *P* = 0.017; OS: 57 months vs. not reached. *P* = 0.023), Similarly, in the patients who did not receive ASCT, the PFS and OS of the CPC high subgroup were significantly shorter than those of the CPC low subgroup (PFS:10 months vs. 40 months, *P* = 0.001; OS:58 months vs. not reached. *P* = 0.041) (Fig. 2).

To assess the effect of CPC number on survival in patients with different genetic hit numbers. We regrouped the patients with FISH no abnormalities, single-hit or double/multi-hit into subgroups according to CPC high or CPC low, and the result showed that the outcome of the CPC high subgroup was significantly poorer than that of the CPC low subgroup in FISH no abnormalities, single-hit or double/multi-hit group (*P* < 0.05) (For details, see Supplementary Fig. 2).

Furthermore, we conducted univariate and multivariate Cox model analysis: LDH > 250 IU/L (HR = 3.130, 95% CI 1.292–4.501, *P* = 0.017), CPC high (HR = 2.351, 95% CI 1.275–3.608, *P* = 0.001), and no ASCT (HR = 2.907, 95% CI 1.761–4.808, *P* = 0.001) were independent risk factors for a shorter PFS. CPC high (HR = 3.708, 95% CI 1.966–6.993, *P* = 0.001) and no ASCT (HR = 2.755, 95% CI 1.222–6.211, *P* = 0.014) were independent risk factors for shorter OS. These results indicated that CPC is an independent risk factor for poor PFS and OS (Tables 3 and 4).

**Table 3:** Risk factors of PFS

Variable	Univariate			Multivariate		
	HR	95% CI	*P* value	HR	95% CI	*P* value
age ≥ 65 year	2.030	1.301–2.778	0.004	1.409	0.922–2.155	0.113
CPC ≥ 0.05%	2.079	1.455–2.967	0.017	2.351	1.275–3.608	0.001
ISS stage III	1.159	0.801–1.677	0.434			
R-ISS stage III	1.558	1.064–2.283	0.123			
CA high risk	1.639	1.149–2.336	0.546			
LDH ≥ 250IU/L	2.604	1.821–3.731	0.017	3.130	1.292–4.501	0.017
R2-ISS III / IV	2.243	2.006–2.537	0.044	1.199	0.927–1.553	0.168
No ASCT	4.069	2.089–7.927	0.001	2.907	1.761–4.808	0.001

**Table 4:** Risk factors of OS

Variable	Univariate			Multivariate		
	HR	95% CI	*P* value	HR	95% CI	*P* value
age ≥ 65 year	2.364	1.385–4.035	0.002	1.284	0.685–2.404	0.435
CPC ≥ 0.05%	5.060	2.827–9.056	0.001	3.708	1.966–6.993	0.001
ISS stage III	2.075	1.134–3.187	0.037	1.215	0.891–2.517	0.487
R-ISS stage III	2.202	1.489–3.256	0.001	1.830	0.961–3.482	0.066
CA high risk	2.619	1.480–4.637	0.001	1.481	0.728–3.010	0.278
LDH ≥ 250IU/L	2.330	1.311–4.140	0.004	1.185	1.007–2.530	0.148
R2-ISS III / IV	2.072	1.484–2.793	0.001	1.321	0.717–2.433	0.372
No ASCT	3.504	2.041–7.874	0.001	2.755	1.222–6.211	0.014

### R2-ISS staging and the survival

**Fig. 3:**
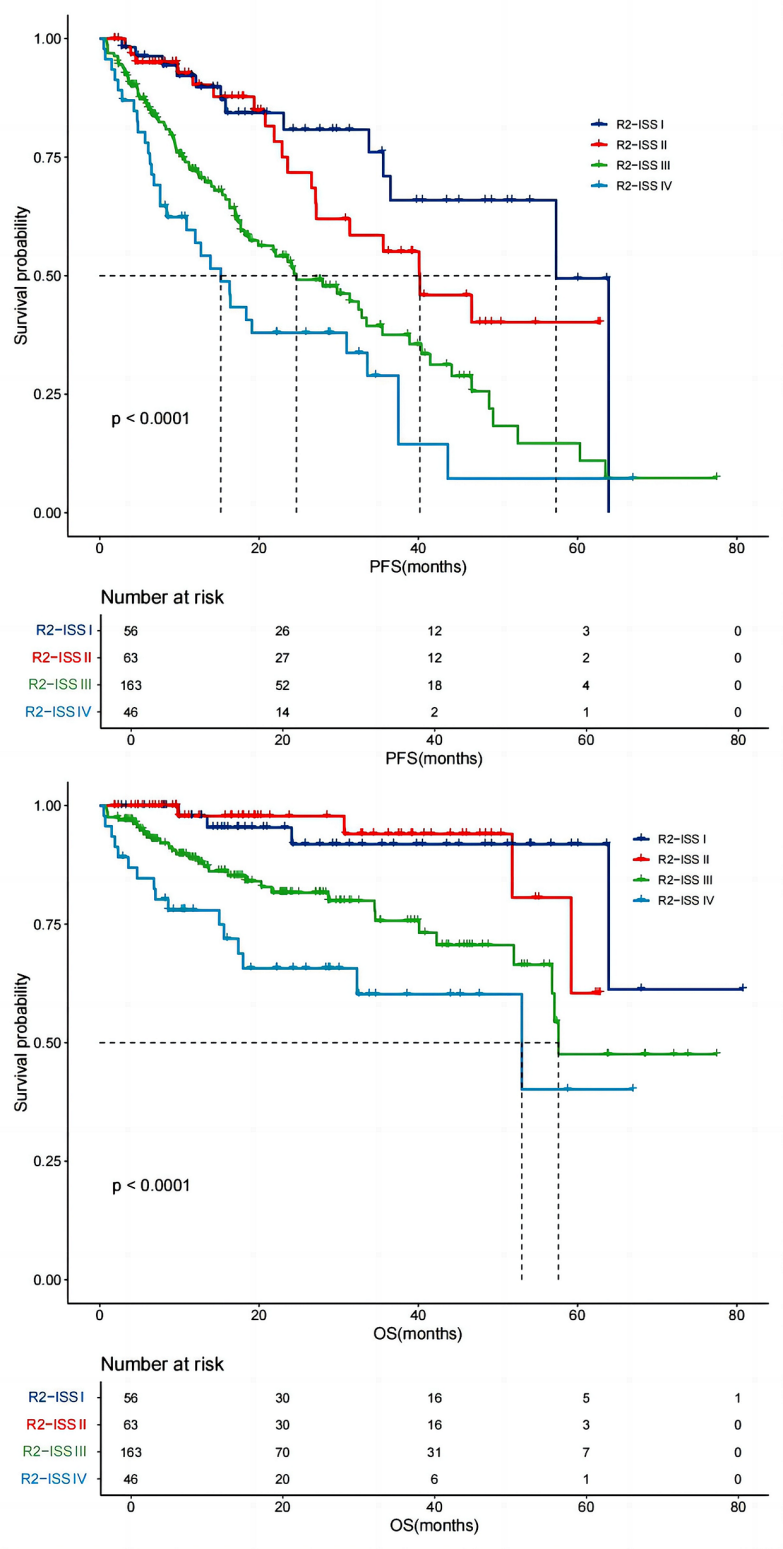
R2-ISS Stage and survival of NDMM patients. Patients were categorized into four groups based on the R2-ISS staging system: stage I-IV. The median PFS for each group was 57 months, 40 months, 25 months, and 15 months, respectively. Median OS were not reached for stages I and II, 58 months for stage III, and 53 months for stage IV

The median PFS for R2-ISS stage I (*n* = 56), stage II (*n* = 63), stage III (*n* = 163) and stage IV (*n* = 46) were 57 months (IQR 24.00–63.70), 40 months (IQR 18.79–46.94), 25 months (IQR 11.66–31.36) and 15 months (IQR 6.47–18.43), respectively. The PFS was significantly longer in patents with R2-ISS stage I than those with stage III (*P* = 0.0001) or with stage IV (*P* = 0.0008). But there was no significant difference in PFS between patients with stage I and stage II (*P* = 0.239) or between patients with stage III and stage IV (*P* = 0.604). The median OS for each group were NR, NR, 58 months (IQR 37.16-NR) and 53 months (IQR 36.72-NR). The OS was significantly longer in patents with R2-ISS stage I than that those with stage III (*P* = 0.0001) or stage IV (*P* = 0.0001). But there was no significant difference in OS between stage I and stage II (*P* = 0.309) or between stage III and stage IV (*P* = 0.391) (Fig. 3).

### Effect of CPC level on survival of NDMM patients with different R2-ISS stage

**Fig. 4:**
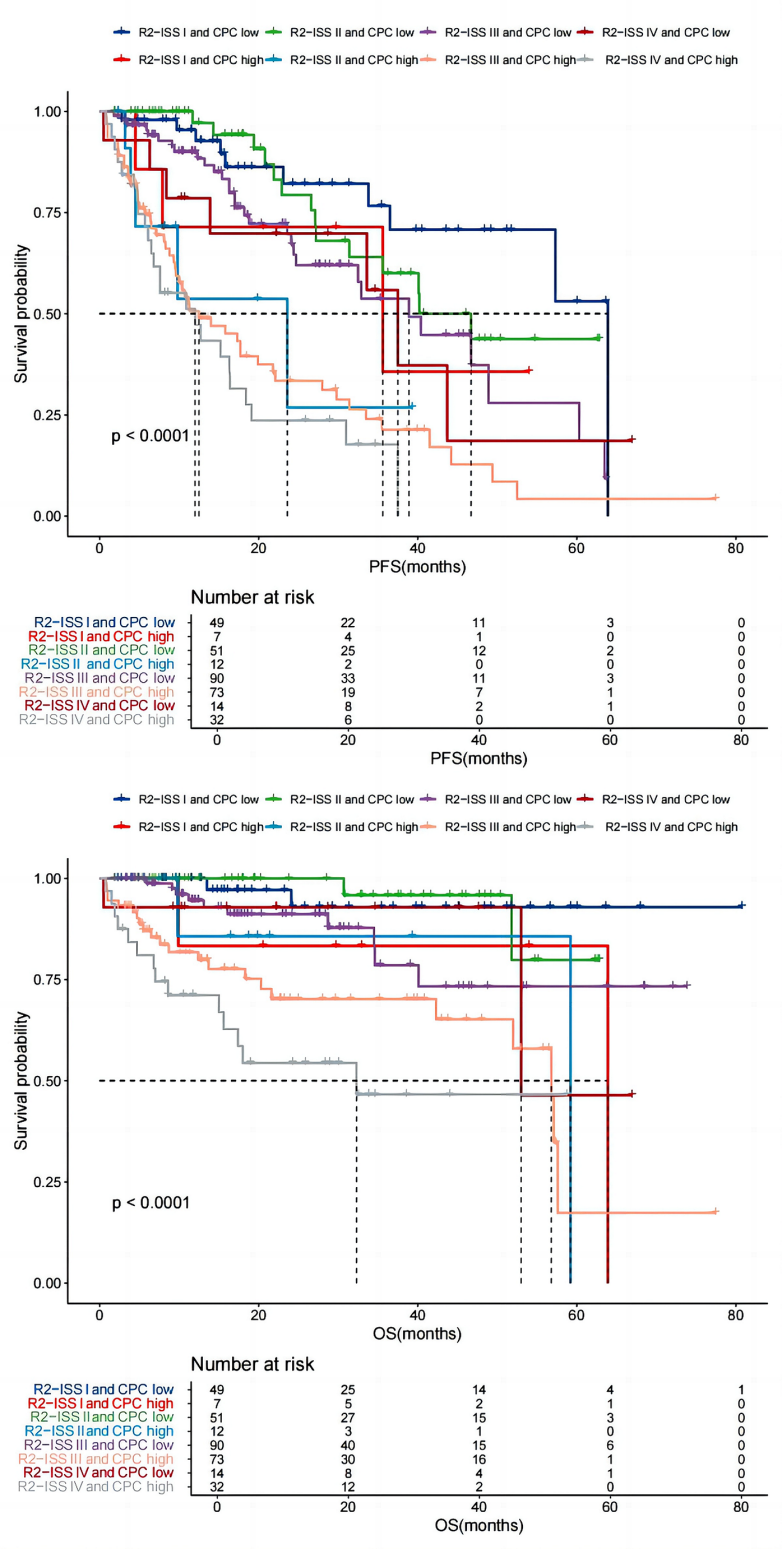
Effect of CPC number on the survival of patients with different R2-ISS stages. Patients with different R2-ISS stages were further stratified based on CPC low or CPC high. All patients were grouped into 8 groups: R2-ISS stage I and CPC low, R2-ISS stage I and CPC high, R2-ISS stage II and CPC low, R2-ISS stage II and CPC high, R2-ISS stage III and CPC low, R2-ISS stage III and CPC high, R2-ISS stage IV and CPC low, and R2-ISS stage IV and CPC high. The median PFS in these 8 groups were 64 months, 36 months, 47 months, 24 months, 39 months, 13 months, 38 months and 12 months, respectively, while the median OS were not reached, 64 months, not reached, 59 months, not reached, 57 months, 53 months, and 32 months, respectively

After adding CPC number to R2-ISS stage, patients were divided into eight groups: R2-ISS I and CPC low (*n* = 49), R2-ISS I and CPC high (*n* = 7), R2-ISS II and CPC low (*n* = 51), R2-ISS II and CPC high (*n* = 12), R2-ISS III and CPC low (*n* = 90), R2-ISS III and CPC high (*n* = 73), R2-ISS IV and CPC low (*n* = 14), and R2-ISS IV and CPC high (*n* = 32). The median PFS in these 8 groups were 64 months (IQR 28.00-NR), 36 months (IQR 20.77–64.22), 47 months (IQR 14.43-NR), 24 months (IQR 18.81–44.32), 39 months (IQR 18.40-NR), 13 months (IQR 8.40–27.53), 38 months (IQR 13.76–45.76) and 12 months (IQR 6.13–20.16). The median OS in these 8 groups were NR, 64 months (IQR 59.17-NR), NR, 59 months (IQR 40.06-NR), NR, 57 months (IQR 20.30–59.57), 53 months (IQR 50.97-NR) and 32 months (IQR 7.03–41.76) (Fig. 4).

There was no significant difference in PFS between patients in stage I with CPC low and patients in stage I with CPC high ( *P* = 0.103), and so was OS ( *P* = 0.095). There was no significant difference in PFS between patients in stage I with CPC high and patients in stage II with CPC low ( *P* = 0.414), and so was OS ( *P* = 0.429). PFS of patients in stage II with CPC low was significantly longer than that of patients in stage II with CPC high ( *P* = 0.001), and so was OS ( *P* = 0.035). PFS of patients in stage II with CPC high was significantly shorter than that of patients in stage III with CPC low ( *P* = 0.038), while no difference was seen in OS between the two groups ( *P* = 0.405). PFS of patients in stage III with CPC low was significantly longer than that of patients in stage III with CPC high ( *P* = 0.001), and so was OS ( *P* = 0.003). There was no significant difference in PFS between patients in stage III with CPC high and that of patients in stage IV with CPC low ( *P* = 0.057), and so was OS (*P* = 0.177). PFS of patients in stage IV with CPC low was significantly longer than that of patients in stage IV with CPC high ( *P* = 0.010), and so was OS ( *P* = 0.048) (Fig. 4).

### A new model by combining R2-ISS stage and CPC level for risk stratification

**Fig. 5:**
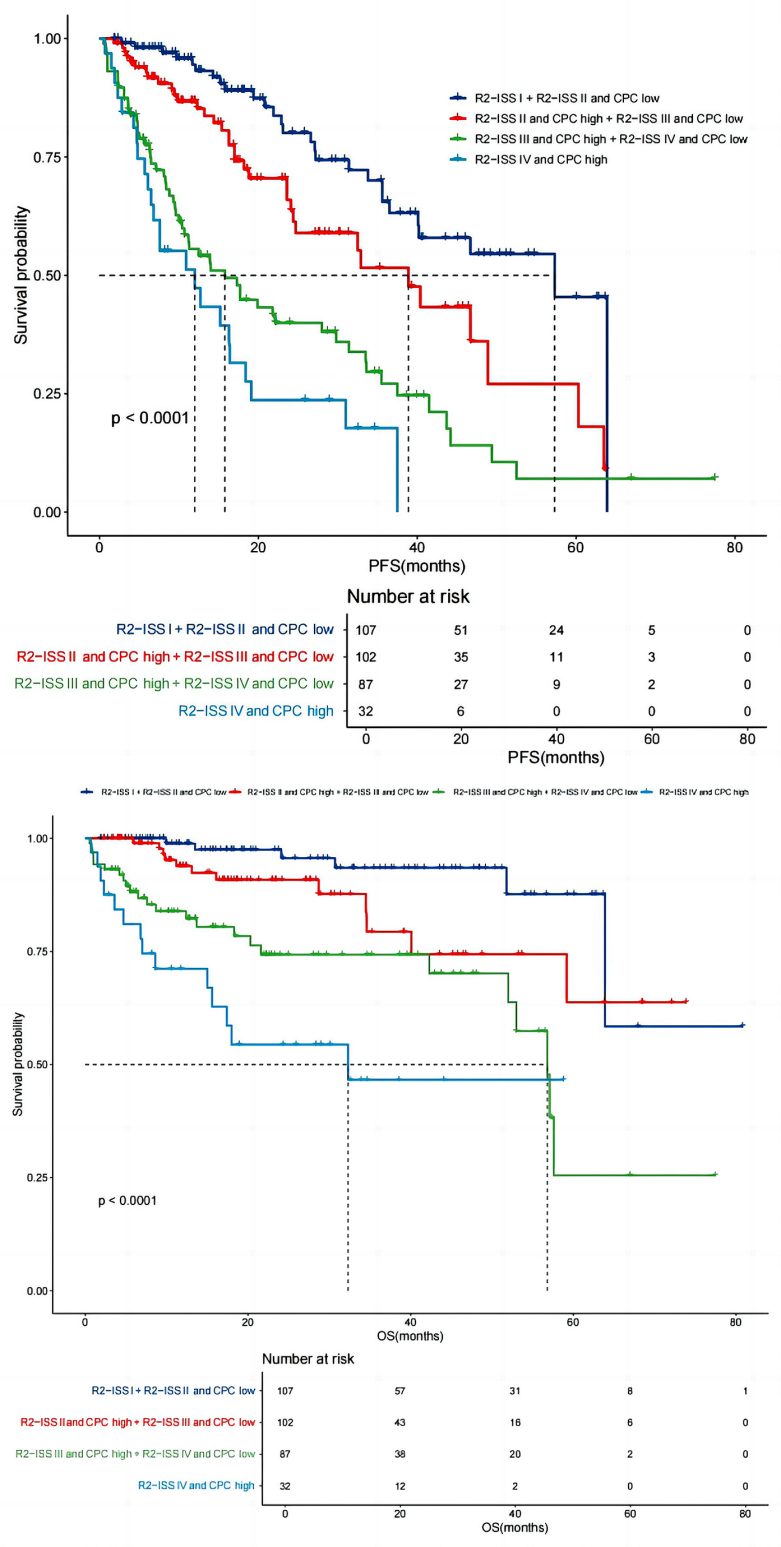
New risk grouping after combined CPC number and R2-ISS Staging. By combining CPC numbers with R2-ISS staging all the patients were regrouped into four different risk categories: group 1: R2-ISS stage I + R2-ISS stage II and CPC low, group 2: R2-ISS stage II and CPC high + R2-ISS stage III and CPC low, group 3:R2-ISS stage III and CPC high + R2-ISS stage IV and CPC low, group 4 : R2-ISS stage IV and CPC high. The median PFS in these 4 groups were 57 months, 39 months, 16 months and 12 months, respectively, while the median OS were not reached, not reached, 57 months and 32 months, respectively

Patients with similar OS were combined and so four new distinct risk groups were formed: Group 1: R2-ISS stage I + R2-ISS stage II and CPC low (*n* = 107), Group 2: R2-ISS stage II and CPC high + R2-ISS stage III and CPC low (*n* = 102), Group 3: R2-ISS stage III and CPC high + R2-ISS stage IV and CPC low (*n* = 87), and Group 4: R2-ISS stage IV and CPC high (*n* = 32). The median PFS of patients in Group 1–4 were 57 months (IQR 14.30–65.56), 39 months (IQR 17.49–44.34), 16 months (IQR 8.49–28.68) and 12 months (IQR 5.36–18.61). The median OS were NR, NR, 57 months (IQR 21.57–61.82) and 32 months (IQR 7.03–40.60). PFS of Group 1 was significantly longer than that of Group 2 (*P* = 0.009). There was no significant difference in PFS between Group 3 and group 4 (*P* = 0.094). OS of Group 1 was significantly longer than that of Group 2 (*P* = 0.033). OS in Group 2 was significantly longer than that of Group 3 (*P* = 0.007). OS in Group 3 was significantly longer than that of Group 4 (*P* = 0.041) (Fig. 5).

## Discussion

With an enhanced comprehension of tumor biology and the emergence of new drugs, the prognosis of MM patients has markedly improved, resulting in prolonged OS. Nonetheless, the disease remains incurable, particularly for individuals exhibiting high-risk characteristics [1]. In this study, we propose the combination of R2-ISS staging and CPC count, which presents an improved risk stratification model.

Previous research has indicated that no single biomarker can fully capture the clinical outcome variants in MM patients [2]. Despite established prognosis prediction systems such as ISS and R-ISS, the highly heterogeneous outcomes in MM patients are not fully explained. The new prognostic stratification model, R2-ISS, developing from clinical trial data can divide NDMM patients into four significantly different groups particularly addressing the issue of survival heterogeneity in R-ISS stage II [5]. However, in several real-world studies [6–10], consistent conclusions were drawn that there were no significant differences in outcome between patients with R2-ISS stage I and stage II. As for survival of patients with R2-ISS stage III and stage IV, conclusions were inconsistent. Tan JLC et al [7] reported that no difference between these two groups. This conclusion is consistent with ours. Contrarily, another study [10] reported that patients with R2-ISS stage III and stage IV represent two groups with different prognosis. Additionally, we found that the proportion of patients with R2-ISS stage III increased significantly compared to R-ISS stage III (39–58% vs. 14–25%) in the same cohort in previous studies [6–10]. In this current study, 50% cases were of R2-ISS stage III. In the EMN population [5] the proportion were 41.2%. Thus, a new problem may arise: how to solve the internal heterogeneity of prognosis in patients with R2-ISS stage III.

Higher number of CPC may portend a more aggressive biological behavior [23]. Previous studies [24–28] have consistently demonstrated CPC level can serve as a prognostic factor for MM patients, correlating to faster disease progression and poorer OS. The NGF technique can detect CPC in 92% of NDMM patients [15] although it is rarely utilized in clinical practice in China. In our study, CPC were detected in 56% of cases in the cohort by widely used 8 color flow cytometry. Our new model is able to distinguish four groups with different outcomes, especially to identify the worst group with OS less than 3 years (Group 4), which cannot be identified by the R2-ISS staging alone. Moreover, adding CPC partly solved the challenge of distinguishing survival heterogeneity in R2-ISS stages III.

Despite these insightful findings, several limitations should be acknowledged, including its retrospective nature and single-center design. Additionally, the study primarily focuses on patients treated with IMiD and PI, necessitating further exploration of this model to patients treated with other new drugs.

In conclusion, we proposed a new model by combination of R2-ISS staging and CPC level that offers an improved risk stratification for NDMM patients.This new model is of great practical value in the developing countries.

### Electronic supplementary material

Below is the link to the electronic supplementary material.

Supplementary Material 1

Supplementary Material 2

Supplementary Material 3

## Data Availability

The data that support the findings of this study are available from the corresponding author.
