# Supplementary figures and images for: R2-ISS staging combined with circulating plasma cells improves risk stratification for newly diagnosed multiple myeloma: a single-center real-world study
Source: Ann Hematol. 2024 Jul 3;103(9):3677–90. doi: 10.1007/s00277-024-05806-9 (PMC11358218; doi:10.1007/s00277-024-05806-9)

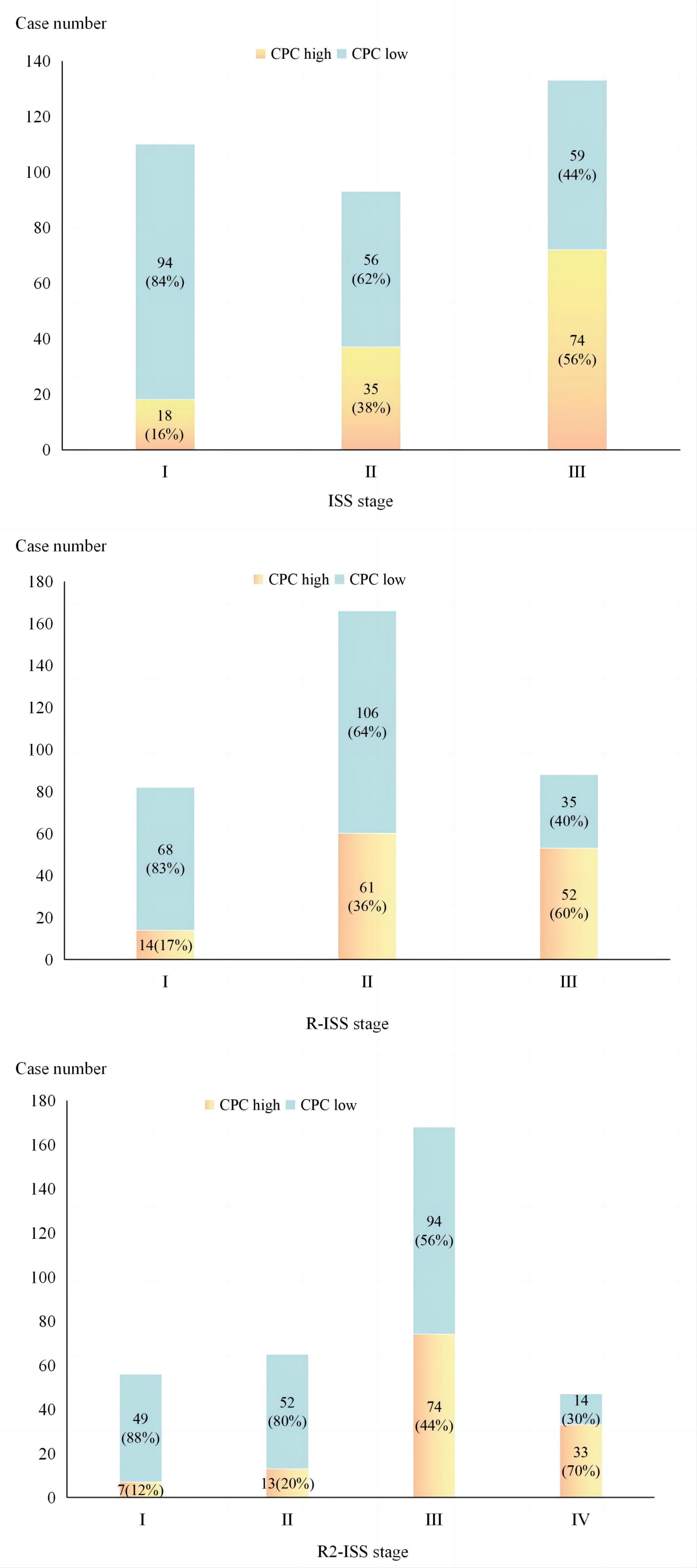

Supplement: Supplementary file 1 — Supplementary Material 1 [file 277_2024_5806_MOESM1_ESM.png]

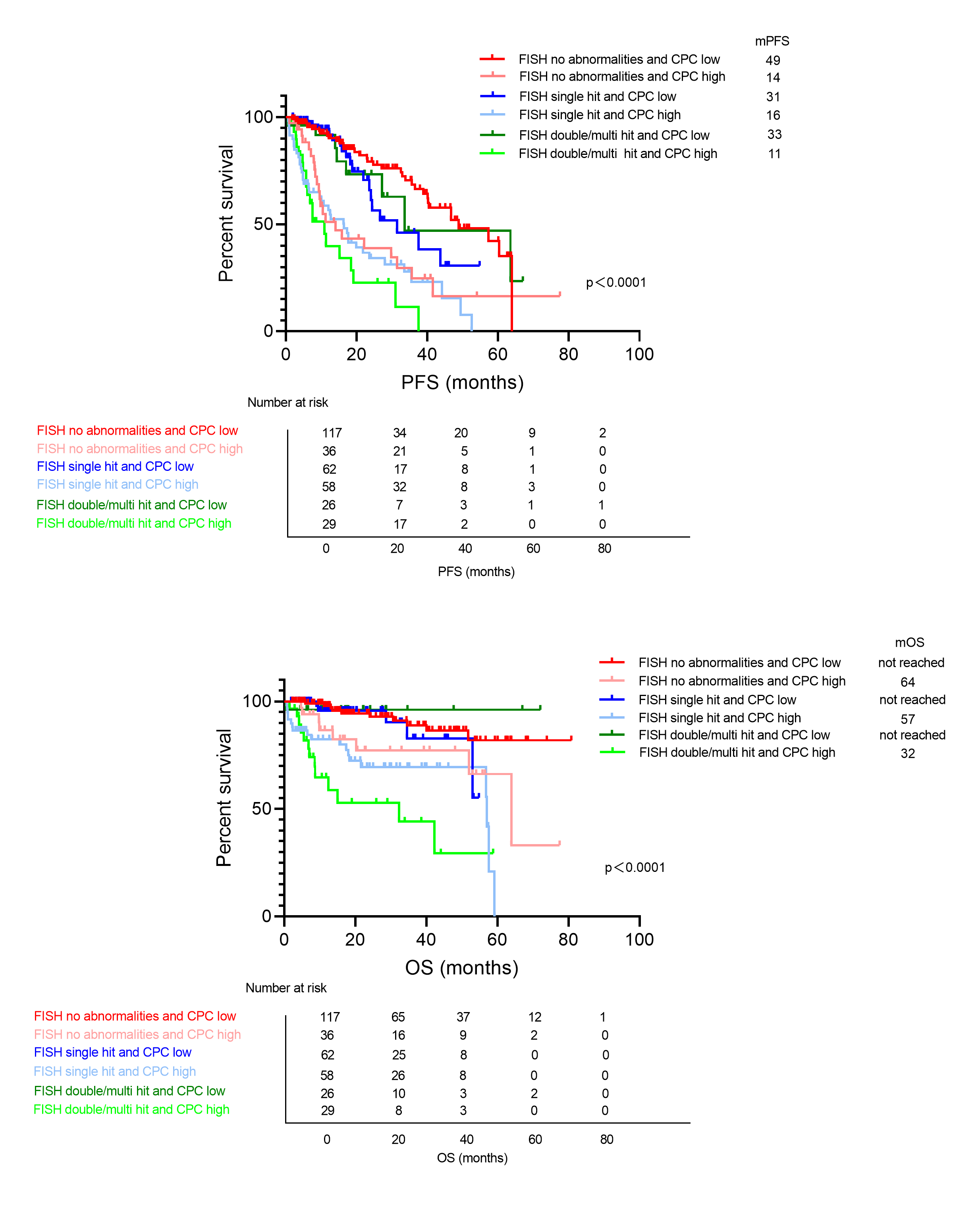

Supplement: Supplementary file 2 — Supplementary Material 2 [file 277_2024_5806_MOESM2_ESM.png]
