# Supplementary material for: R2-ISS staging combined with circulating plasma cells improves risk stratification for newly diagnosed multiple myeloma: a single-center real-world study
Source: Ann Hematol. 2024 Jul 3;103(9):3677–90. doi: 10.1007/s00277-024-05806-9 (PMC11358218; doi:10.1007/s00277-024-05806-9)
Supplement: Supplementary file 3 — Supplementary Material 3 [file 277_2024_5806_MOESM3_ESM.docx]

Supplementary Figure 1: CPC count in different clinical stages in NDMM patients. In ISS stage I-III, the proportion of patients with CPC high in each stage was 16%, 38% and 56%. In R-ISS stage I-III, the proportions of patients with CPC high in each stage were 17%, 36% and 60%. In R2-ISS stage I-IV , the proportions of patients with CPC high in each stage were 12%, 20%, 44% and 70%.

Supplementary Figure 2 : The effect of CPC count on survival in patients with different genetic hit numbers. We divided the patients with FISH no abnormalities, single-hit or double/multi-hit into 6 subgroups according to CPC high or CPC low. group 1: FISH no abnormalities and CPC low, group 2: FISH no abnormalities and CPC high, group 3: FISH single hit and CPC low, group 4 : FISH single hit and CPC high. group 5: FISH double/multi hit and CPC low, group 6 : FISH double/multi hit and CPC high. The median PFS in these 6 groups were 49 months, 14 months, 31 months, 16 months, 33 months and 11 months, respectively, while the median OS were not reached, 64 months, not reached, 57 months, not reached and 32 months, respectively.
